# Supplementary material for: Association between oral microbiome and seven types of cancers in East Asian population: a two-sample Mendelian randomization analysis
Source: Front Mol Biosci. 2023 Nov 21;10:1327893. doi: 10.3389/fmolb.2023.1327893 (PMC10702768; doi:10.3389/fmolb.2023.1327893)
Supplement: Supplementary file 1 [file DataSheet1.ZIP › Supplementary Materials/MR plots for tongue/tongue═╝/Colorectal cancer/pheno.860_to_colorectal cancer_forest.pdf]

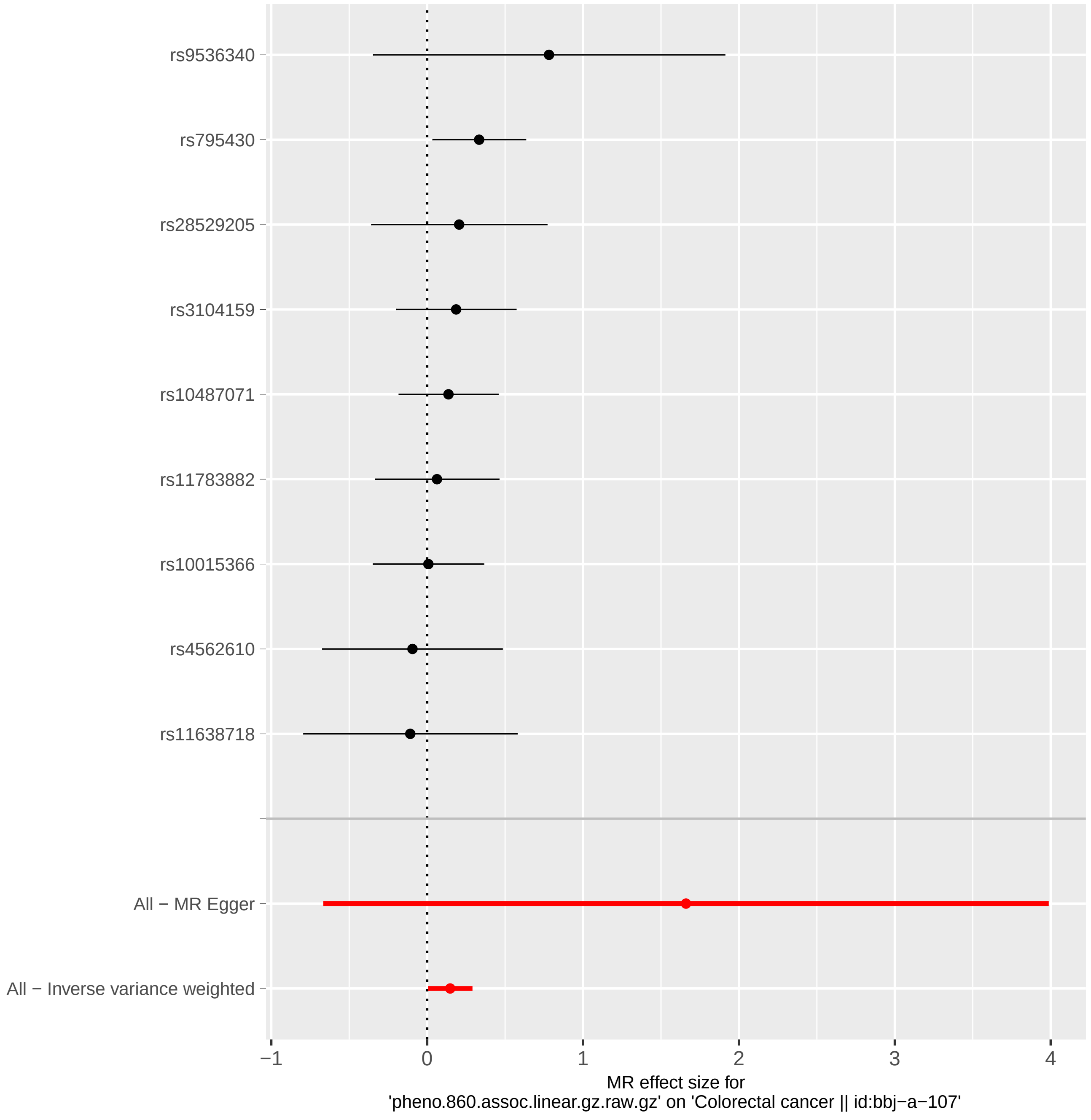

rs9536340
rs795430
rs28529205
rs3104159
rs10487071
rs11783882
rs10015366
rs4562610
rs11638718
All − MR Egger
All − Inverse variance weighted
−1
0
1
2
3
4
MR effect size for
'pheno.860.assoc.linear.gz.raw.gz' on 'Colorectal cancer || id:bbj−a−107'
